# Effects of Aerobic Exercise Training on the Growth, Swimming Performance, Antipredation Ability and Immune Parameters of Juvenile Rock Carp (*Procypris rabaudi*)

**DOI:** 10.3390/ani12030257

**Published:** 2022-01-21

**Authors:** Qimiao Hou, Shijian Fu, Tiji Huang, Xiuming Li, Xiaotao Shi

**Affiliations:** 1 Laboratory of Evolutionary Physiology and Behavior, Chongqing Key Laboratory of Animal Biology, Chongqing Normal University, Chongqing 400047, China; 2019110513054@stu.cqnu.edu.cn (Q.H.); shijianfu9@cqnu.edu.cn (S.F.); 2021110513067@stu.cqnu.edu.cn (T.H.); 2 Hubei International Science and Technology Cooperation Base of Fish Passage, China Three Gorges University, Yichang 443002, China

**Keywords:** aerobic exercise training, growth, swimming performance, antipredation ability, immune parameters, *Procypris rabaudi*

## Abstract

**Simple Summary:**

The artificial release of hatchery fish has not achieved expected results, mainly due to the low survival rate of the fish after being released into the natural environment. Many studies have shown that the implementation of pro-released training can improve the survival rate of hatchery fish. Rock carp (*Procypris rabaudi*) is a rare breed of fish in the upper and middle reaches of the Yangtze River. The aim of this study is to investigate whether aerobic exercise training has positive effects on the growth, swimming ability, antipredation ability and immunologic function before releasing the species into the wild. The findings from this study indicate that the training regime at close to 2–4 bl s^−1^ for 20 h per day for 42 days prior to release might be a suitable strategy to promote the survival rate of released juvenile rock carp in the wild.

**Abstract:**

Many studies have found that aerobic exercise training at a moderate water velocity can improve the growth, swimming performance and survival rate of fish. To investigate the effects of aerobic exercise training on the growth, swimming performance, antipredation ability and immune parameters of rock carp, juveniles were placed in training channels with different water velocities (i.e., 3 cm s^−1^, 1 (body length s^−1^) bl s^−1^, 2 bl s^−1^ and 4 bl s^−1^) for 6 weeks. Then, the specific growth rate, critical swimming speed (*U*_crit_) and its metabolism, constant acceleration speed (*U*_cat_), survival rate under predation, spleen index, lysozyme (LZM) activity and immunoglobulin (IgM) level were measured. Training showed no significant effect on the length-specific growth rate, weight-specific growth rate, *U*_crit_, maximum metabolic rate (MMR), metabolic scope (MS), *U*_cat_ or spleen index. The resting metabolic rates (RMRs) of the 2 bl s^−1^ and 4 bl s^−1^ training groups were significantly higher than those of the control group and 1 bl s^−1^ training group. The survival rate of the 1 bl s^−1^ training group in the presence of predators was significantly higher than that of the control group but significantly lower than those of the 2 bl s^−1^ and 4 bl s^−1^ training groups. The LZM activity of the 4 bl s^−1^ training group was significantly higher than that of the control group. The IgM level of the 2 bl s^−1^ training group was significantly higher than that of the control group. These data indicate that aerobic exercise training does not improve the growth and swimming performance of juvenile rock carp but can improve their antipredation ability and immunologic function.

## 1. Introduction

The growth, reproduction and survival of fish are significantly affected by various environmental factors in nature, such as temperature, food and predators [1,2,3]. Due to the alternation of the dry season and rainy season and the construction of artificial water conservation facilities, the water velocity in a fish habitat often changes significantly. The fluctuation in water current can stimulate the sensory and motor organs of fish, which generate corresponding modes of motion and rejection mechanisms and have an influence on the tissue morphology, exercise physiology and individual behavior of fish [4,5,6]. Fish often can swim against a water current and make ideal subjects for studies on the effects of exercise training [7]. According to the demand for oxygen, the movement of fish can be divided into two types: aerobic and anaerobic [8]. Therefore, exercise training protocols have been categorized as aerobic or anaerobic in fish [6,7,9]. Aerobic exercise training usually occurs in a continuous, uninterrupted manner. The duration ranges from 2 weeks to 1 year, and the energy for aerobic metabolism is mainly provided by red muscles during training [7,10,11]. Studies have found that aerobic exercise training can have significant effects on the growth performance, spontaneous behavior and swimming ability of fish [7,12,13,14].

As a way of escaping, predating and breeding, swimming performance is important for the survival of fish and is an important parameter that can be used to evaluate the adaptability of fish to the natural environment and to design fishways [3,15,16,17]. Critical swimming speed (*U*_crit_) is an important indicator that is widely used to determine the aerobic exercise ability of fish and is closely related to their activities, such as foraging and migrating [2,15,16,18]. Constant acceleration speed (*U*_cat_) is an important index widely used to evaluate the anaerobic exercise ability of fish, which is closely related to their ability to swim in rapids and avoid predators [16,19,20]. Normally, the swimming abilities of fish have strong plasticity. Numerous studies have found that the swimming performances of many kinds of fish, such as common carp (*Cyprinus carpio*), darkbarbel catfish (*Pelteobagrus vacheli*), qingbo (*Spinibarbus sinensis*) and bream (*Megalobrama pellegrini*), have been significantly improved after exercise training [6,21,22,23]. More importantly, it has been found that improved swimming performance can enhance antipredation capacity and thus improve the survival rate of individuals [23]. In addition, the immune parameters of fish are closely related to their survival rate, so they have received extensive attention [24,25,26]. Many researchers have found that proper exercise can clearly promote the immune parameters of fish, but the training effect is closely linked to the kinds of fish and training parameters, such as type, intensity and duration [25,26,27,28,29,30,31].

Rock carp (*Procypris rabaudi*) is a species of bony fish in the family Cyprinidae. This species lives in the cracks between rocks at the bottom of the Yangtze River with some natural predators such as snakehead (*Channa argus*) and southern catfish (*Silurus* *meridionalis*). It is a rare breed of fish in the upper and middle reaches of the Yangtze River [32]. In recent years, the wild population of this species has diminished because of overfishing and habitat destruction. In the *China Red Book of Endangered Animals (fish)*, it is an endangered species [33]. Meanwhile, it has a relatively low swimming ability in cyprinid fish and is an important kind of fish for proliferation and release in the field [34]. A previous study found that anaerobic exercise training (exhaustive chasing training, once daily for 21 days) had negative impacts on the growth performance and the resting metabolic rate (RMR) of the species [35]. Therefore, the aim of this study is to investigate whether aerobic exercise training has positive effects on growth, swimming ability, antipredation ability and immunologic function before releasing the endangered species into the wild. To achieve these aims, we assessed the specific growth rate (SGR), *U*_cat_, *U*_crit_ and metabolic rate, survival under predation, spleen index, LZM activity and IgM level in juvenile rock carp after aerobic exercise training under different water velocities.

## 2. Materials and Methods

### 2.1. Experimental Animals

Juvenile rock carp were obtained from a fisheries hatchery (Yongchuan, Chongqing, China) and were acclimated in a recirculating system (250 L) for 4 weeks prior to the experiment. The fish were fed twice a day (09:00 and 18:00) to satiation using commercial food (Tongwei Company, Sichuan, composition: 41.2 ± 0.9% protein, 8.5 ± 0.5% lipid, 25.7 ± 1.2% carbohydrate and 12.3 ± 0.4% ash). The predators, i.e., snakehead (*Channa argus*) (*n* = 12, 200–300 g, 25–29 cm), were also obtained from the same fisheries hatchery and fed to satiation with cutlets of freshly killed loach once daily (10:00) in another recirculating system (250 L). Uneaten food and feces were removed with a siphon 1 h after feeding. During this period, the water temperature was controlled at 25 ± 1 °C, and the oxygen content of the water was maintained above 7.0 mg L^−1^. A third of the total water volume was replaced daily, and the photoperiod was 12 h light–12 h dark.

### 2.2. Aerobic Exercise Training Protocol

At the end of the acclimation period, 240 fish of similar size ((3.95 ± 0.06) g, (6.30 ± 0.03) cm) were randomly divided into a control group and 1 bl s^−1^ (body length per second), 2 bl s^−1^ and 4 bl s^−1^ training groups, and they were placed in four separate channels of the self-developed “Fish Swimming Sports Training Instrument” [12]. The instrument consists of a water-processing and temperature-controlling system and a tank (length, 190 cm; width, 110 cm; depth, 25 cm) containing four training channels. Continuous water velocities in the experimental channel were achieved via the motors (30 w) with a propeller. Different water velocities were produced by controlling the different voltages of transducer power. Each group (60 fish, 3 replicates and 20 fish per replicate) was evenly divided into 3 cells (20 fish per cell) of a channel. The control group was placed in a channel with a water velocity of 3 cm s^−1^. The 1 bl s^−1^, 2 bl s^−1^ and 4 bl s^−1^ groups were trained for 20 h each day at the corresponding training water velocity, and the aerobic training lasted for 6 weeks. The rearing conditions during training were the same as those during the acclimation period.

### 2.3. Parameter Measurements

#### 2.3.1. Measurement of Growth Rate

Before and after the training, the body weight of the fish was weighed by electronic scales (accurate to 0.1 g), and the body length (standard length) of the fish was measured by a ruler (accurate to 0.1 cm). The growth rate of the experimental fish was calculated as follows:

Weight-specific growth rate (%/d) = 100 × (LnW2 − LnW1)/T(1)

Length-specific growth rate (%/d) = 100 × (LnL2 − LnL1)/T(2)

where W1 and W2 are the body weights of the experimental fish before and after aerobic exercise training, respectively; L1 and L2 are the body lengths of the experimental fish before and after aerobic exercise training, respectively; and T is the duration of the experiment (42 days).

#### 2.3.2. Measurement of *U*_cat_

*U*_cat_ was measured by Blazka-type swim tunnel respirometers (volume, 3.5 L) [6]. The fish were individually (*n* = 9, Table 1) transferred into the swim tunnel for 1 h of acclimation, and the water velocity was 6 cm/s (i.e., 1 body length s^−1^). The flow of aerated water through the respirometer was maintained continuously during this recovery period. After acclimation, the water velocity in the swim tunnel continuously increased at a rate of 0.1667 cm s^−2^ (10 cm s^−1^ min^−1^). A computer outputted various electric pulses to the stepping motor of the swimming tunnel respirometer and thus changed the water velocity in the tunnel. The water was accelerated at this rate until the fish were exhausted. The water velocity when the fish were exhausted was the *U*_cat_ value [16,19]. The criterion of exhaustion was that the fish were unable to swim forward and stayed for more than 20 s in the rear honeycomb screen of the swimming chamber [9].

#### 2.3.3. Measurements of U_crit_ and the Metabolic Rate

*U*_crit_ was also determined by Blazka-type swim tunnel respirometers (volume, 3.5 L) [6]. The fish were individually (*n* = 9, Table 1) transferred into the swim tunnel for 6 h of acclimation, and the water velocity was 6 cm/s (i.e., 1 body length s^−1^). After acclimation, the water velocity was increased at an increment of 6 cm/s in closed mode, and the continuous swimming duration at each water speed was 20 min. The experiment was stopped when the experimental fish showed exhaustion. The criterion of exhaustion was that the fish were unable to swim forward and stayed for more than 20 s in the rear honeycomb screen of the swimming chamber [2]. Each fish was analyzed using Brett’s equation to calculate the *U*_crit_:

*U*_crit_ = [V + (T/ΔT) ΔV] (3)

where V is the highest speed at which the fish swam for the full time period (cm s^−1^); T is the duration for which the fish swam at the final speed (min); ΔT is the prescribed period of swimming at that speed (i.e., 20 min); and ΔV is the velocity increment (i.e., 6 cm/s).

The metabolic rate (MO_2_) of each fish while swimming was measured during the measurement of *U*_crit_. The specific purpose was to measure the dissolved oxygen value of the water body close to the oxygen meter every 2 min. The absolute value of the slope of the dissolved oxygen value over time and the volume of the water were used to calculate the metabolic rate of each experimental fish during the exercise. At the end of the experiment, the fish were removed, the swimming metabolometer was closed, and the oxygen consumption rate was measured for 20 min to eliminate the influence of bacterial oxygen consumption on the MO_2_ of the experimental fish. The calculation formula for MO_2_(mgO_2_ kg^−1^ h^−1^)) was as follows:

MO_2_ = (St − S_0_) × 60 × 3.45/m (4)

where St is the slope of the dissolved oxygen value changing with time during each shift (20 min), S_0_ is the slope of the dissolved oxygen value changing with bacteria oxygen consumption, 3.45 is the volume of the swimming channel (L), 60 is 60 min and m is the body weight (kg) of the fish. The maximum observed O_2_ during the *U*_crit_ test was defined as the maximum metabolic rate (MMR). The RMR was estimated by exponentially fitting O_2_ as a function of steady swimming speeds and extrapolating back to speed zero, while the metabolic scope (MS) was the difference between the MMR and RMR.

#### 2.3.4. Measurement of the Immune Parameters

Six fish from each group (i.e., two fish per cell, Table 1) were randomly selected under mild anesthesia (MS−222, 50 mg/L). The body weight of the fish was weighed with an electronic balance (accurate to 0.0001 g), and the body length of the fish was measured with a ruler (accurate to 0.1 cm). Then, the spleen of the fish was removed and weighed (accurate to 0.0001 g).

Spleen index (‰) = spleen weight/body weight × 1000(5)

Twelve fish from each group (i.e., four fish per cell) were sampled to determine immune parameters. The fish were rapidly placed in water containing a concentration of anesthetic (MS−222, 50 mg/L, Sigma-Aldrich, St Louis, MO, USA). The tail of the fish was cut off, and blood samples were collected immediately. Following centrifugation (3000 r/min for 10 min), the serum was separated and stored at −20 °C for the determination of the LZM activity and IgM level. The LZM activities were determined according to the turbidimetric method [36] using commercial test kits (Nanjing Jiancheng Institute, Nanjing, China). The IgM levels were measured according to the enzyme-linked immunosorbent assay (ELISA) [37] using commercial test kits (Nanjing Jiancheng Institute, Nanjing, China).

#### 2.3.5. Measurement of Antipredation Ability

To measure the antipredation ability of juvenile rock carp, sixteen fish from each group were selected and tagged with a visible implant elastomer and allowed to recover for 24 h. All predators were fasted for 24 h prior to the experiment. Four fish (16 in total, Table 1) were selected from each of the four groups, and two predators (body length, 25–29 cm; body weight, 200–300 g) were placed together in the experimental water tank (length, 80 cm; width, 80 cm; height, 50 cm; volume, 200 L). The experiment was observed every 30 min and lasted between 20 and 30 h. The experiment was stopped when half of the fish in the experimental water tank were eaten by the predators. The experiments were repeated in four identical tanks, i.e., *n* = 4 [9].

### 2.4. Data Analysis

The experimental data were calculated by Excel and analyzed by SPSS 17.0. The effects of aerobic exercise training on all parameters were analyzed by one-way ANOVA. The effects of aerobic exercise training and water velocity on the metabolic rate were assessed using a two-way ANOVA. The ANOVA was followed by a least-significant-difference multiple-comparison test when appropriate. Differences were considered significant at *p* < 0.05, and values were presented as the mean (±SE).

## 3. Results

### 3.1. Growth Performance

There were no significant differences in terminal body weight, terminal body length, specific body weight growth rate or specific body length growth rate between the training groups (1, 2 and 4 bl s^−1^) and the control group (Table 2).

### 3.2. Swimming Ability and Metabolic Rate

**Figure 1:**
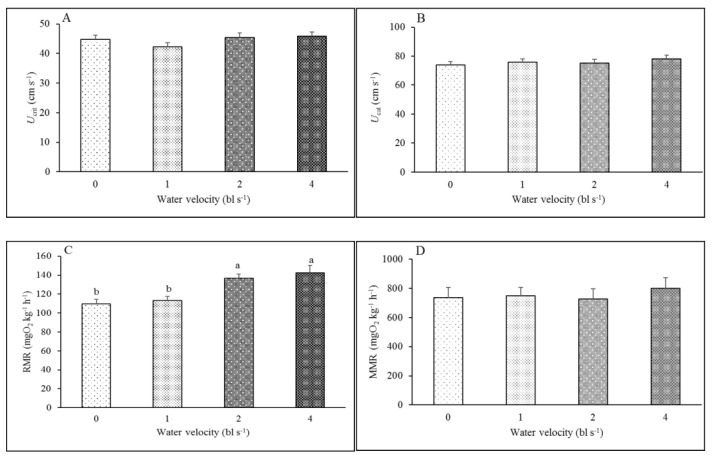

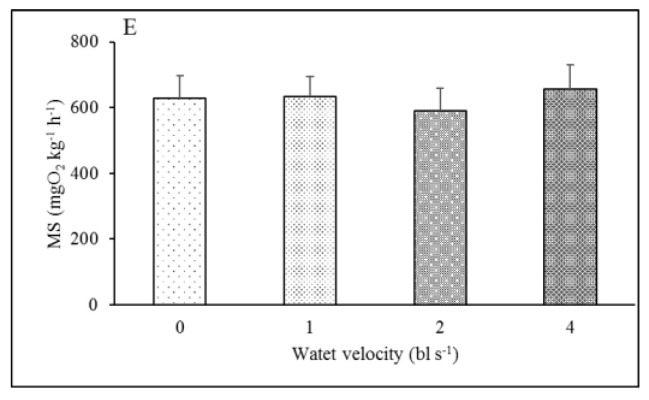
The effect of aerobic exercise training on *U*_crit_, *U*_cat,_ RMR, MMR and MS in juvenile rock carp (mean ± S.E., *n* = 9). (**A**). *U*_crit_; (**B**). *U*_cat_; (**C**). RMR; (**D**). MMR; (**E**). MS. Values without a common lowercase letter indicate a significant difference.

**Figure 2:**
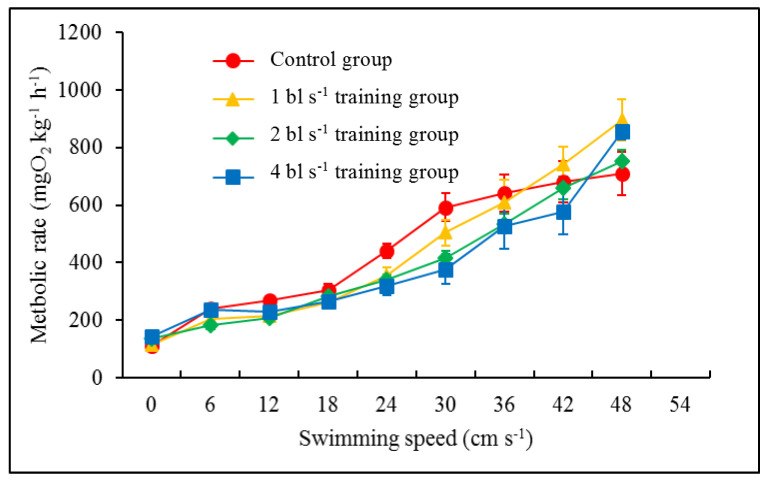
The effects of swimming speed on the oxygen consumption rate (MO_2_) in juvenile rock carp (mean ± S.E., *n* = 9) at different water velocities.

There were no significant differences in *U*_crit_ (44.80, 42.33, 45.33 and 45.93 cm s^−1^), *U*_cat_ (74.08, 75.82, 75.13 and 78.23 cm s^−1^), MMR (737.87, 747.75, 727.89 and 799.49 mgO_2_ kg^−1^ h^−1^) or MS (627.99, 634.73, 591.09 and 656.97 mgO_2_ kg^−1^ h^−1^) between the control group and the training groups (1, 2 and 4 bl s^−1^) (Table 3, Figure 1). The RMRs in the 2 bl s^−1^ and 4 bl s^−1^ training groups (136.79 and 142.52 mgO_2_ kg^−1^ h^−1^, respectively) were significantly higher than those in the control group and the 1 bl s^−1^ training group (109.87 and 113.02 mgO_2_ kg^−1^ h^−1^, respectively) (*F*_3,35_ = 8.330, *p* < 0.001) (Table 3, Figure 1C). With increasing water velocity, the metabolic rate of juvenile fish in each group significantly increased (*F*_7,265_ = 83.262; *p* < 0.001) (Figure 2 and Table 4).

### 3.3. Antipredation Ability

**Figure 3:**
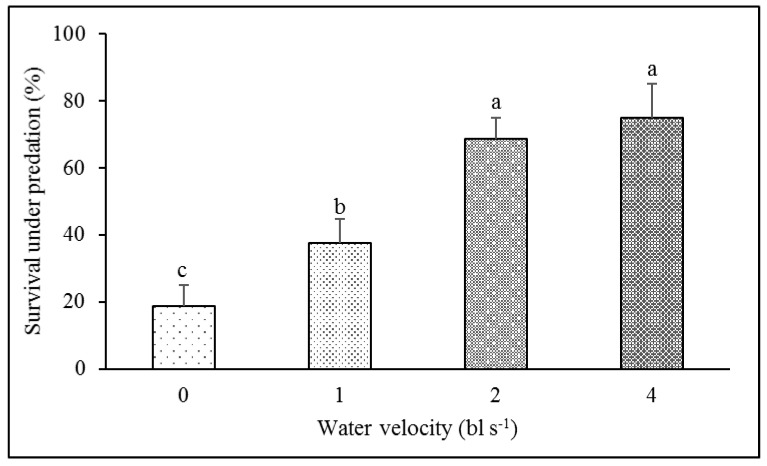
The effect of aerobic exercise training on survival under predation in juvenile rock carp (mean ± S.E., *n* = 4). Values without a common lowercase letter indicate a significant difference.

The survival rate of the fish in the 1 bl s^−1^ training group (37.50%) was significantly higher than that in the control group (18.75%) in the presence of predators. There was no significant difference in the survival rates between the 2 bl s^−1^ and 4 bl s^−1^ training groups (68.75 and 75.00%, respectively) in the presence of predators. However, the survival rates of the 2 bl s^−1^ and 4 bl s^−1^ training groups were significantly higher than those of the 1 bl s^−1^ training group and the control group (*F*_3,15_ = 8.407, *p* = 0.003) (Table 3, Figure 3).

### 3.4. Immune Parameters

**Figure 4:**
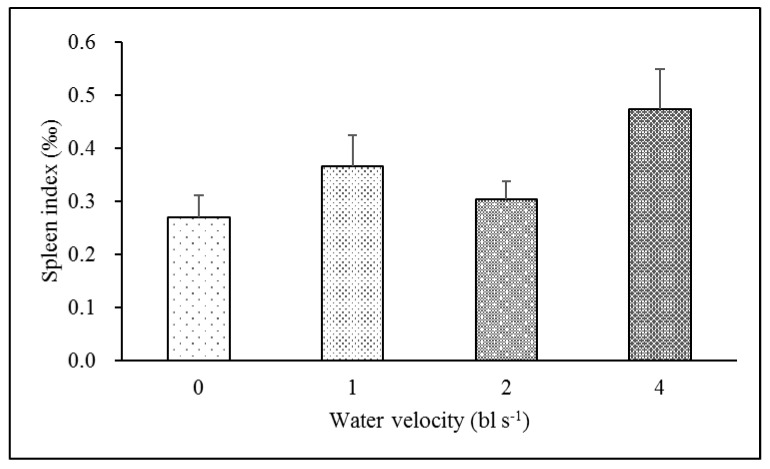
The effect of aerobic exercise training on spleen index in juvenile rock carp (mean ± S.E., *n* = 6). Values without a common lowercase letter indicate a significant difference.

**Figure 5:**
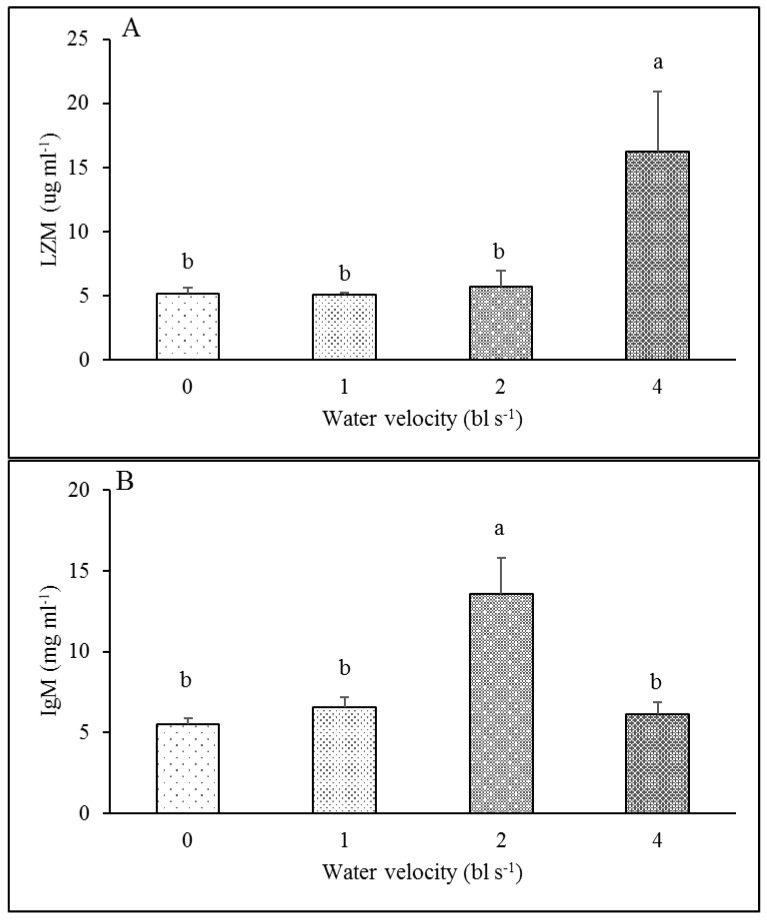
The effect of aerobic exercise training on the LZM activity and IgM level in juvenile rock carp (mean ± S.E., *n* = 6). (**A**). LZM; (**B**). IgM. Values without a common lowercase letter indicate a significant difference.

There was no significant difference in the spleen index between the training groups (0.37, 0.30 and 0.47‰, respectively) and the control group (0.27‰) (Table 3, Figure 4). The LZM activity of the 4 bl s^−1^ training group (16.23 μg mL^−1^) was significantly higher than these of the other three groups (5.16, 5.05 and 5.73 μg mL^−1^, respectively) (*F*_3,23_ = 4.629, *p* = 0.014) (Table 3, Figure 5A). The IgM level of the 2 bl s^−1^ training group (13.59 mg mL^−1^) was significantly higher than those of the other three groups (5.50, 6.58 and 6.12 mg mL^−1^, respectively) (*F*_3,23_ = 9.547, *p* < 0.001) (Table 3, Figure 5B)

## 4. Discussion

### 4.1. Effect of Aerobic Exercise Training on the Growth of Juvenile Rock Carp

The effects of exercise training on fish growth are related to the training methods, training duration, training equipment and water velocity [7,12,22,30]. Most studies have found that anaerobic exercise usually has a negative impact on the growth of fish [22,35,38,39], while aerobic exercise training at an appropriate water velocity oppositely improves the growth of fish [4,5,28,40]. Moreover, the effect of exercise training on growth performance is also dependent on the lifestyle of fish species [40,41,42]. For example, the growth performances of active swimmers, such as yellowtail (*Seriola quinqueradiata*) [40], the Atlantic salmon (*Salmo salar*) [28] and Arctic charr (*Salvelinus alpinus*) [43], can be improved by aerobic exercise training at a moderate water velocity. In contrast, aerobic exercise training had negative effects on growth performances of some inactive swimmers, such as the rabbitfish (*Siganus rivulatus*) [42] and the Japanese flounder (*Paralichthys olivaceus*) [41]. However, this study found that aerobic training had no significant effect on the growth ability of juvenile rock carp. This finding suggests that the growth capacity of juvenile rock carp did not benefit from continuous aerobic exercise, which may be related to the inactive lifestyle of the species.

### 4.2. Effect of Aerobic Training on Swimming Performance

Numerous studies have found that appropriate continuous aerobic training can improve the critical swimming speed of many fish, such as common carp [21] trained at 60% *U*_crit_ speed for 28 days, black carp (*Mylopharyngodon piceus*) [44] trained at 2 and 4 bl s^−1^ water velocity for 8 weeks and bream [6] trained at 4 bl s^−1^ water velocity for 5 weeks. However, this study found that *U*_crit_ was not significantly different among the control and training groups. This result means that continuous aerobic training did not improve the swimming performance of the juvenile rock carp. This situation has been previously documented in fish species such as the leopard shark (approximately 0.7 bl s^−1^ for 6 weeks) [45] and chinook salmon (*Oncorhynchus tshawytscha*) (*U*_crit_ swim test on alternate days for 4 weeks) [46]. Some studies have found that the improvement in *U*_crit_ from training fish is accompanied by an improvement in aerobic metabolism [22,47]. MMR and MS are widely accepted evaluation indexes of aerobic metabolic performance [6]. In this study, the difference in the MMR and MS between the training group and the control group was not obvious. This scenario may be one reason why aerobic training did not improve the *U*_crit_ of the rock carp.

Aerobic exercise training can affect the body shape and physical characteristics of fish and reduce phosphocreatine, the release of catecholamine and cortisol and the rate of lactate clearance in muscle and blood. These factors may affect the anaerobic exercise and metabolic ability of fish [3,7,48,49]. However, there are few reports about aerobic training on the anaerobic exercise and metabolic ability of fish. A recent study found that continuous aerobic training can improve the anaerobic exercise ability of *Schizothorax wangchiachii* (10 cm/s for 6 and 12 h per day for 30 days) [10]. In this study, the results showed that there was no significant difference in *U*_cat_ between the control group and the training group (6 weeks of training at a velocity of 1–4 bl s^−1^). These results show that continuous aerobic training did not improve the anaerobic exercise ability of the juvenile rock carp. Therefore, the influence of aerobic training on anaerobic exercise ability may be related to the species and training system of the fish. More study on the effects of aerobic training on the anaerobic exercise ability of fish with different habits and physiological and biochemical mechanisms may be conducive to revealing the internal causes of anaerobic exercise ability.

### 4.3. Effect of Aerobic Exercise Training on Antipredation Ability

One of the main purposes of fish exercise training research is to improve the survival rate of fish in the wild [7]. It has been found that the survival rate of chinook salmon and Atlantic salmon can be improved significantly through exercise training [50]. Short-term swimming training before release to the wild helps brown trout (*Salmo trutta*) obtain a much higher survival rate than the control group [51]. Furthermore, the survival rate of Chinese suckers (*Myxocyprinus asiaticus*) subjected to anaerobic exercise training was significantly reduced when attacked by predators [9]. Therefore, we expect aerobic exercise training to improve the antipredator ability of fish. As expected, we found that in the presence of predators, the survival rate of the training group (6 weeks at a velocity of 1–4 bl s^−1^) was notably higher than that of the control group, which indicates that continuous aerobic exercise training has noticeably enhanced the antipredator ability of juvenile rock carp. The *U*_crit_ and *U*_cat_ are closely related to the survival rate of fish in the presence of predators [23]. However, aerobic exercise training did not improve the *U*_crit_ and *U*_cat_ of juvenile rock carp. Those results suggested that the improved antipredation ability may not be related to the swimming performance of the species after aerobic exercise training. We found that the resting metabolic rate of the juvenile carp was remarkably higher than that of the control group, and these experimental data suggest that continuous aerobic exercise training obviously boosts the minimum energy demand of juvenile carp in maintaining fluid circulation, protein synthesis and osmotic pressure regulation; may shorten the emergency response time; and improves its ability to identify predators. This may be one of the reasons why the survival rate of the juvenile rock carp increased notably in the presence of predators after aerobic exercise training.

### 4.4. Effect of Exercise Training on Immune Response

The immune organ index is an important indicator reflecting the degree of immune organ development and is positively correlated with immune function in healthy fish [52]. Lysozyme is mainly found in neutrophils and monocytes, with lower activities also present in macrophages, and plays a key role in the nonspecific immune response in fish [31,53]. IgM actively performs many functions such as agglutination complement activation, enhancing cellular-mediated cytotoxicity, and neutralization of pathogens and is the primary immunoglobulin essential for defense mechanisms in fish [54,55]. Exercise training can have a significant impact on the immune parameters of fish, but its training effect is closely related to its training mode [26,30,31]. Anaerobic exercise training often has negative influences on the immune parameters of different kinds of fish. For example, exhaustion training (once daily or twice daily, 3 weeks) resulted in a decrease in LZM activity for qingbo [26]. However, a large number of studies have found that aerobic exercise training has a positive effect on the LZM activity of fish, such as tinfoil barb (*Barbodes schwanenfeldii*) (0.1 and 0.3 m/s, 45 days) [25], Prenant’s schizothoracin (*Schizothorax prenanti*) (1, 2 (bl) s^−1^, 8 weeks) [30] and tinfoil barb (0.66 bl/s, and 1.92 bl/s, 23 and 45 days) [31]. Aerobic exercise training (2 bl/s and 4 bl/s, 8 weeks) led to a significant increase in IgM level for qingbo [26]. In this study, LZM activity (4 bl s^−1^) and IgM level (2 bl s^−1^) of the training groups were significantly higher than those of the control group. These results indicated that continuous aerobic training (2–4 bl s^−1^) had a positive effect on the immune functions of the juvenile rock carp. Furthermore, this study found that the LZM activity of the 4 bl s^−1^ training group was the highest; however, the IgM level of the 2 bl s^−1^ training group was the highest after aerobic exercise training. The results may be due to the different effects of aerobic exercise training at the same water velocity on the different immune parameters of fish species. For example, aerobic exercise training at 2 and 4 bls ^−1^ improved IgM levels for qingbo. However, this training regime did not have a significant effect on LZM activity of the species [26].

## 5. Conclusions

In conclusion, this study found that aerobic training had no obvious effect on the growth performance of the juvenile rock carp. Furthermore, after aerobic training, the exercise ability of the juvenile rock carp was not significantly improved, which may be related to the fact that the MMR and MS were not greatly improved. However, aerobic exercise training (2 and 4 bl s^−1^) profoundly improved the antipredation ability of the juvenile rock carp, which may have been due to the significant shortening of its emergency response time with a consequently increased maintenance metabolic expenditure [23,56]. At the same time, aerobic exercise training at 2 and/or 4 bl s^−1^ also significantly improved the immune indexes, such as those of LZM and IgM, of the juvenile rock carp, which means that aerobic exercise training is beneficial for improving its immune function. Thus, aerobic exercise training at close to 2–4 bl s^−1^ for 20 h per day for 42 days prior to release appears to be a suitable regime for antipredation ability and immune function enhancement, thus potentially increasing the survivability of released juvenile rock carp in the wild.

## Figures and Tables

**Table 1:** Body mass and body length of the experimental fish in the present study (mean ± S.E.).

Parameters		Water Velocity			
		Control	1 bl s^−1^	2 bl s^−1^	4 bl s^−1^
*U*_crit_					
Body mass (g)	*n* = 9	4.47 ± 0.15	4.25 ± 0.18	4.07 ± 0.14	4.22 ± 0.28
Body length (cm)	*n* = 9	6.66 ± 0.09	6.67 ± 0.12	6.43 ± 0.11	6.48 ± 0.09
*U*_cat_					
Body mass (g)	*n* = 9	4.45 ± 0.10	4.54 ± 0.26	4.05 ± 0.16	4.57 ± 0.23
Body length (cm)	*n* = 9	6.62 ± 0.07	6.53 ± 0.11	6.53 ± 0.08	6.72 ± 0.08
Survival rate					
Body mass (g)	*n* = 4	4.38 ± 0.16	3.77 ± 0.17	3.69 ± 0.17	3.15 ± 0.09
Body length (cm)	*n* = 4	6.44 ± 0.08	6.26 ± 0.08	6.19 ± 0.08	6.01 ± 0.14
LZM					
Body mass (g)	*n* = 6	4.13 ± 0.17	4.84 ± 0.18	4.40 ± 0.12	3.99 ± 0.29
Body length (cm)	*n* = 6	6.45 ± 0.13	6.71 ± 0.09	6.31 ± 0.07	6.15 ± 0.18
IgM					
Body mass (g)	*n* = 6	4.40 ± 0.32	5.07 ± 0.39	4.18 ± 0.36	3.75 ± 0.19
Body length (cm)	*n* = 6	6.51 ± 0.13	6.70 ± 0.19	6.37 ± 0.18	6.18 ± 0.09
Spleen					
Body mass (g)	*n* = 6	4.23 ± 0.15	4.08 ± 0.28	4.52 ± 0.06	4.92 ± 0.54
Body length (cm)	*n* = 6	6.58 ± 0.09	6.48 ± 0.13	6.60 ± 0.07	6.90 ± 0.09

**Table 2:** The effect of aerobic exercise training on the growth performance of juvenile rock carp (mean ± S.E., *n* = 3).

Parameters	Training				Results of One-Way ANOVA
	Control Group	1 bl s^−1^ Training Group	2 bl s^−1^ Training Group	4 bl s^−1^ Training Group	Training Effect
Initial body weight (g)	4.12 ± 0.23	4.04 ± 0.32	3.83 ± 0.20	3.82 ± 0.27	*F*_3,11_ = 0.068; *p* = 0.396
Initial body length (cm)	6.36 ± 0.09	6.29 ± 0.11	6.32 ± 0.07	6.23 ± 0.09	*F*_3,11_ = 0.368; *p* = 0.788
Final body weight (g)	4.38 ± 0.04	4.31 ± 0.28	4.08 ± 0.19	4.20 ± 0.11	*F*_3,11_ = 0.518; *p* = 0.681
Final body length (cm)	6.53 ± 0.06	6.52 ± 0.13	6.36 ± 0.08	6.41 ± 0.09	*F*_3,11_ = 0.022; *p* = 0.800
Length-specific growth rate (% d^−1^)	0.08 ± 0.02	0.09 ± 0.04	0.05 ± 0.03	0.08 ± 0.05	*F*_3,11_ = 0.362; *p* = 0.782
Weight-specific growth rate (% d^−1^)	0.18 ± 0.13	0.19 ± 0.08	0.18 ± 0.08	0.28 ± 0.18	*F*_3,11_ = 0.179; *p* = 0.907

*n* = 3 replicates, 20 fish per replicate.

**Table 3:** The effect of aerobic exercise training on several variables related to swimming performance and immune function in juvenile rock carp based on the results of one-way analysis of variance (ANOVA).

Parameters	Covariate Effect	Training Effect
Critical swimming speed (*U*_crit_)	*F*_1,35_ = 13.745, *p* = 0.001	*F*_3,35_ = 2.127, *p* = 0.117
Constant acceleration speed (*U*_cat_)	*F*_1,35_ = 1.150, *p* = 0.292	*F*_3,35_ = 0.445, *p* = 0.723
Resting metabolic rate (RMR)	*F*_1,35_ = 0.031, *p* = 0.860	*F*_3,35_ = 8.330, *p* < 0.001
Maximum metabolic rate (MMR)	*F*_1,35_ = 9.620, *p* = 0.004	*F*_3,35_ = 0.479, *p* = 0.699
Metabolic scope (MS)	*F*_1,35_ = 10.220, *p* = 0.003	*F*_3,35_ = 0.245, *p* = 0.864
Survival under predation	*F*_1,15_ = 1.697, *p* = 0.219	*F*_3,15_ = 8.407, *p* = 0.003
LZM	*F*_1,23_ = 0.303, *p* = 0.588	*F*_3,23_ = 4.629, *p* = 0.014
IgM	*F*_1,23_ = 1.067, *p* = 0.315	*F*_3,23_ = 9.547, *p* < 0.001
Spleen index	*F*_1,23_ = 8.151, *p* = 0.010	*F*_3,23_ = 1.945, *p =* 0.157

**Table 4:** The effect of aerobic exercise training on the metabolic rate of juvenile rock carp under different water velocities (mean ± S.E.).

Parameters		Training				Results of Two-Way ANOVA		
		Control Group	1 bl s^−1^ Training Group	2 bl s^−1^ Training Group	4 bl s^−1^ Training Group	Velocity Effect	Training Effect	Interaction Effect
Metabolic rate (mg O_2_ kg^−1^ h^−1^)	6 cm s^−1^	239.15 ± 4.57 ^Ca^	203.78 ± 8.51 ^Dab^	180.95 ± 14.22 ^Gb^	236.93 ± 15.71 ^Da^	*F*_7,265_ = 83.262; *p <* 0.001	*F*_3,265_ = 4.490; *p* = 0.004	*F*_21,265_ = 1.287; *p* = 0.185
	12 cm s^−1^	267.27 ± 13.14 ^Cb^	215.08 ± 10.57 ^Da^	206.20 ± 10.32 ^Ga^	228.25 ± 10.85 ^Da^			
	18 cm s^−1^	303.11 ± 22.49 ^Ca^	262.61 ± 13.70 ^CDa^	283.02 ± 10.54 ^Fa^	264.20 ± 16.61 ^CDa^			
	24 cm s^−1^	439.12 ± 24.84 ^Bb^	355.75 ± 27.61 ^Ca^	340.54 ± 18.81 ^Ea^	317.48 ± 29.67 ^CDa^			
	30 cm s^−1^	592.48 ± 47.96 ^Ac^	503.90 ± 44.1 ^Bbc^	414.52 ± 26.42 ^Dab^	377.45 ± 50.72 ^Ca^			
	36 cm s^−1^	640.93 ± 65.21 ^Aa^	609.56 ± 76.59 ^Ba^	534.31 ± 33.81 ^Ca^	525.07 ± 78.09 ^Ba^			
	42 cm s^−1^	679.92 ± 71.63 ^Aa^	743.45 ± 58.74 ^Aa^	659.74 ± 39.38 ^Ba^	577.67 ± 78.71 ^Ba^			
	48 cm s^−1^	708.89 ± 75.65 ^Aa^	895.67 ± 72.71 ^Aa^	751.12 ± 39.69 ^Aa^	856.23 ± 28.69 ^Aa^			

Values in each row without a common lowercase letter indicate a significant difference. Values in each column without a common capital letter indicate a significant difference.

## Data Availability

Data are available upon request from the corresponding author.
